# Shift work is significantly and positively associated with dementia: A meta-analysis study

**DOI:** 10.3389/fpubh.2023.998464

**Published:** 2023-02-16

**Authors:** Kuo-Wei Lee, Chen-Cheng Yang, Chun-Hung Chen, Chih-Hsing Hung, Hung-Yi Chuang

**Affiliations:** ^1^Department of Neurology, Kaohsiung Municipal Siaogang Hospital, Kaohsiung Medical University, Kaohsiung, Taiwan; ^2^Department of Neurology, Kaohsiung Medical University Hospital, Kaohsiung Medical University, Kaohsiung, Taiwan; ^3^College of Medicine, Graduate Institute of Medicine, Kaohsiung Medical University, Kaohsiung, Taiwan; ^4^Department of Occupational and Environmental Medicine, Kaohsiung Municipal Siaogang Hospital, Kaohsiung Medical University, Kaohsiung, Taiwan; ^5^Department of Occupational and Environmental Medicine, Kaohsiung Medical University Hospital, Kaohsiung Medical University, Kaohsiung, Taiwan; ^6^Research Center for Precision Environmental Medicine, Kaohsiung Medical University, Kaohsiung, Taiwan; ^7^Environmental and Occupational Medicine Center, Kaohsiung Municipal Siaogang Hospital, Kaohsiung Medical University, Kaohsiung, Taiwan; ^8^Department of Public Health and Environmental Medicine, Kaohsiung Medical University, Kaohsiung, Taiwan

**Keywords:** shift work, night shift, occupational medicine, meta-analysis, dementia, Alzheimer disease

## Abstract

**Background:**

Shift work may disrupt the sleep and wake cycles and negatively affect physical and mental health. Dementia is a neurodegenerative disorder with progressively declining cognition that is receiving increasing attention. Studies on the association between shift work and dementia are rare. Herein, we conducted a meta-analysis to investigate the association between shift work and dementia.

**Materials and methods:**

This study was conducted according to the Preferred Reporting Items for Systematic Reviews and Meta-Analyses guidelines. We searched the PubMed, Embase, and Web of Science databases using a related set of keywords. The inclusion criteria were as follows: (1) adult employees working in a factory, company, or organization; (2) exposure to shift work/non-shift work; and (3) outcome of dementia based on examination or assessment. A meta-analysis using a fixed-effects model was performed. The hazard ratio of dementia was compared between shift workers and non-shift workers.

**Results:**

Five studies were included in the quantitative synthesis, and two were selected for further meta-analysis. A random-effects model showed a modest association between shift work and an increase in dementia cases (pooled hazard ratio = 1.13; 95% confidence interval: 1.04–1.23; *p* = 0.04). This association also occurred in night workers for more than 1 year.

**Conclusion:**

Shift work and long-term night work were modestly associated with a higher risk of developing dementia. Avoiding long-term night shifts may be effective in reducing dementia risk. Further studies are required to confirm this hypothesis.

## Introduction

Shift work, which is generally defined as a work schedule comprised of irregular or unusual hours compared with a normal daytime work schedule, may disrupt the sleep and wake cycle and have a negative effect on health (1). A previous study reported an increase in all-cause mortality among shift workers (2). Shift work is not only associated with poor physical health but also has negative effects on mental health (3).

Dementia, which involves a group of symptoms affecting memory, planning, and social abilities severely enough to interfere with one's daily life, is most common caused by Alzheimer's disease. In a previous study, The Alzheimer's Association has reported that cognitive decline and dementia have non-modifiable risk factors, such as age, family history, and apolipoprotein E ε4 allele genetic polymorphisms, and modifiable risk factors, including traumatic brain injury, midlife obesity, midlife hypertension, diabetes, smoking, dyslipidemia, history of depression, and sleep disturbance (4). Shift work, especially night shift work, disturbed biological circadian rhythms (5). Two prospective cohort studies of around 1,200 community-dwelling women age 65 or older recruited from participants of the Study of Osteoporotic Fractures, have reported that weaker circadian activity rhythm patterns, which defined as in the lowest quartile of circadian activity rhythms amplitude, MESOR (midline estimating statistic of rhythm), or robustness measured by wrist actigraphy, in older woman without dementia are associated with worse cognitive function, especially executive function and increased risk of developing dementia, independent of sleep factors, and a number of comorbidities and health factors (6, 7). Another recent cohort study of 2,764 community-dwelling men aged 65 years and older revealed positive associations between disrupted rest-activity circadian rhythm, which was measured by wrist actigraphy, and cognitive decline after an average of 3.4 years of follow-up (8). However, a cohort study that evaluated midlife shift-work history among 16,190 participants in the Nurses' Health Study reported that there were no associations between shift-work history and cognitive decline (9). Another cross-sectional study randomly invited 425 former and current employees of a German university hospital aged 55 and older to undergo cognitive testing. Though with low response, 47 people (11% of the total sample) completing the cognitive test and revealed the frequency of indication for slight or pronounced impairment did not differ between those working with and without shifts (10). The long-term effects of shift work on cognitive impairment and dementia in later life still showed inconsistent results.

A recent review evaluating the association of shift work and dementia, failed to extrapolate definite conclusions, due to the limited number of available studies and heterogenous results (11). Since shift work disrupts circadian rhythm and circadian rhythm desynchronization may have effect on neurodegeneration. Under the hypothesis, we conducted a comprehensive meta-analysis of current studies to quantify the association between the incidence of dementia and the history of shift work.

## Materials and methods

### Protocol and registration

This study was performed in accordance with the Preferred Reporting Items for Systematic Reviews and Meta-Analyses (PRISMA) guidelines. The review protocol was registered at PROSPERO (Identifier, CRD42022316541) and Kaohsiung Medical University Hospital Institutional Review Broad (KMUHIRB-EXEMPT(I)-20220005).

### Data sources and search terms for study selection

We searched the MEDLINE (PubMed), Embase, and Web of Science databases on January 22, 2022, for related studies. We did not limit the dates of studies published in these databases, and all studies including the target keywords were considered. Two researchers (K–WL and C–CY) performed preliminary searches using different keywords. The researchers separately proposed a set of key search words that were then merged to one final list, as follows: “Schedule, Shift Work” OR “Schedules, Shift Work” OR “Work Schedule, Shift” OR “Night Shift Work” OR “Shift Work, Night” OR “Rotating Shift Work” OR “Shift Work, Rotating” OR “Shift Work Schedule”[Mesh] OR day-time[Title/Abstract] OR night-time[Title/Abstract] OR “Day-time” OR “Night-time” AND “Dementias” OR “Amentia” OR “Amentias” OR “Senile Paranoid Dementia” OR “Dementias, Senile Paranoid” OR “Paranoid Dementia, Senile” OR “Paranoid Dementias, Senile” OR “Senile Paranoid Dementias” OR “Familial Dementia” OR “Dementia, Familial” OR “Dementias, Familial” OR “Familial Dementias” OR “Alzheimer's Disease” OR “Dementia, Senile” OR “Senile Dementia” OR “Dementia, Alzheimer Type” OR “Alzheimer Type Dementia” OR “Alzheimer-Type Dementia (ATD)” OR “Alzheimer Type Dementia (ATD)” OR “Dementia, Alzheimer-Type (ATD)” OR “Alzheimer Type Senile Dementia” OR “Primary Senile Degenerative Dementia” OR “Dementia, Primary Senile Degenerative” OR “Alzheimer Sclerosis” OR “Sclerosis, Alzheimer” OR “Alzheimer Syndrome” OR “Alzheimer Dementia” OR “Alzheimer Dementias” OR “Dementia, Alzheimer” OR “Dementias, Alzheimer” OR “Senile Dementia, Alzheimer Type” OR “Acute Confusional Senile Dementia” OR “Senile Dementia, Acute Confusional” OR “Dementia, Presenile” OR “Presenile Dementia” OR “Alzheimer Disease, Late Onset” OR “Late Onset Alzheimer Disease” OR “Alzheimer's Disease, Focal Onset” OR “Focal Onset Alzheimer's Disease” OR “Familial Alzheimer Disease (FAD)” OR “Alzheimer Disease, Familial (FAD)” OR “Alzheimer Diseases, Familial (FAD)” OR “Familial Alzheimer Diseases (FAD)” OR “Alzheimer Disease, Early Onset” OR “Early Onset Alzheimer Disease” OR “Presenile Alzheimer Dementia” OR “Dementia”[Mesh] OR “Alzheimer Disease”[Mesh]. The search methods for the Embase and Web of Science databases were modified, as appropriate.

### Eligibility criteria

The inclusion criteria for the study were as follows: (1) included adult employees working in a factory/company/organization; (2) assessed exposure to shift work/non-shift work; and (3) provided outcomes of dementia based on examination or assessment.

### Process of article selection

Two researchers (K–W Lee and C–C Yang) independently evaluated the titles and abstracts of the initially identified studies (first round of screening). After removing the duplicates, the full articles were read for those who met the inclusion criteria and those whose eligibility for the title and abstract screening was unclear. Any disagreements between the two researchers were resolved by three other researchers based on discussion and consensus.

### Data collection

From each included study, we extracted information regarding study characteristics, shift work, dementia, and the association between shift work and dementia. We contacted the authors of these for further explanation if the study failed to or imprecisely reported the required data.

### Study characteristics

We obtained the following data regarding study characteristics: publication year, the country where the study was completed, sample size, sampling framework (clinic based, workplace based, or population based), participant characteristics, and estimated risk of outcome (i.e., the hazard ratio [HR] of participants with dementia) where appropriate.

### Shift work

We defined shift work as “work beyond regular working daytime hours,” including evening shift, night shift, fixed shift, on-call shift, or rotating shift (12–15).

### Dementia

The outcome was as follows: definite diagnosis of dementia according to the diagnostic criteria of the Diagnostic and Statistical Manual of Mental Disorders, Fifth Edition (DSM-5) for major neurocognitive disorders.

### Statistical analysis

We computed all pooled HRs from the individual HRs of shift workers and non-shift workers. Standard error (SE) was estimated for the HRs according to the 95% confidence interval (CI). Regarding the main analysis, a random-effects model meta-analysis was used to calculate the pooled HR with 95% CI. We also applied a random-effects model to analyze the possibility of heterogeneity in HRs among these studies, based on their characteristics (16). Among-study heterogeneity was analyzed using *I*^2^. Funnel plots were used to assess publication bias. We also performed a separate meta-analysis of the subgroups of night shifts over 1 year. All analyses were performed using Review Manager version 5.4 and R version 3.6.2.

## Results

### Selected studies

**Figure 1:**
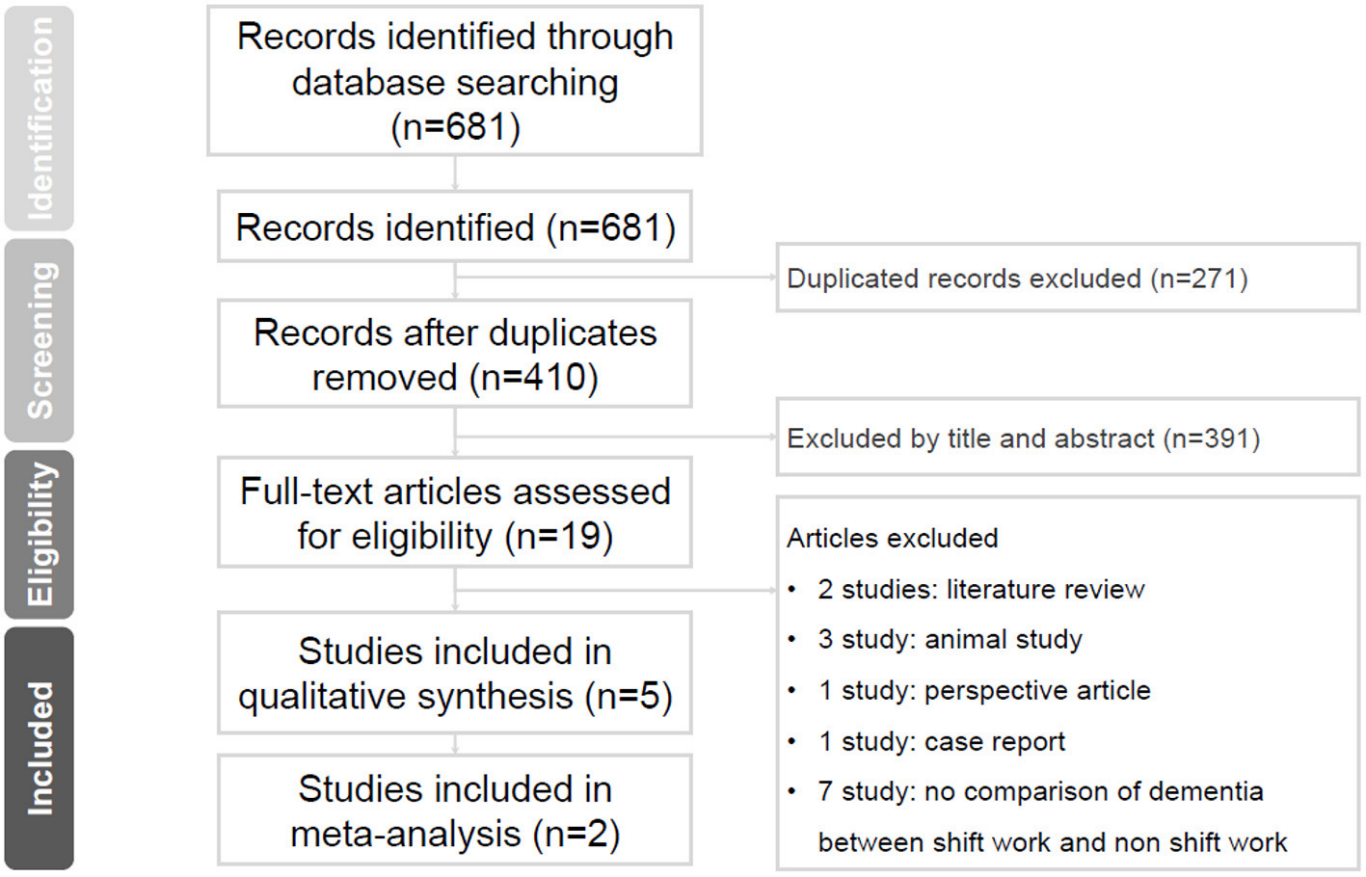
Preferred reporting items for systematic reviews and meta-analyses (PRISMA) flow diagram.

Figure 1 shows the research selection process using the PRISMA flow diagram. An initial database search (PubMed, Embase, and Web of Science) identified 681 articles. Subsequently, 271 duplicates were excluded. After screening the titles and abstracts of 410 studies, 19 articles were considered for a full-text review. After full-article evaluation of the 19 studies, 14 articles were excluded for not meeting the following criteria: no dementia comparison between shift workers and non-shift workers (*N* = 7), systemic review articles (*N* = 2), animal studies (*N* = 3), case reports (*N* = 1), and perspective studies (*N* = 1). Finally, we recruited five studies in the qualitative review and only two studies reporting the hazard ratio of dementia in shift workers were included for further meta-analysis.

### Study characteristics

Table 1 presents details of the five studies that met the inclusion criteria (17–21). All included studies were prospective studies as follow: Nabe-Nielsen et al. (17) (396 and 115 shift with night work and without night work and 2828 non-shift general workers); Jørgensen et al. (21) (11,272, 1805, 980, and 3,958 nurses with day work, evening shift, permanent night shift and rotating shift, respectively); Bokenberger et al. (19) (including two population cohorts: one included 13,283 participants from the Swedish Twin Registry (STR) with 2,258 and 11,025 shift and non-shift workers, the other included 41,199 participants from Screening Across the Lifespan Twin (SALT) study with 12,399 and 28,800 night shift and non-night shift workers); Nabe-Nielsen et al. (20) (1,011 and 3,720 shift and non-shift male workers, respectively); and Jørgensen et al. (18) (11,822, 1,923, 1,048, and 4,099 nurses work on day time, evening shift, night shift and rotating shift, respectively). Two studies estimated incidence rate ratios of dementia in shift workers: one study (20) reported the IRRs for males only while the other study (17) reported sex-combined IRRs. Two studies reported the hazard ratio of dementia in shift workers: one (18) reported the HRs for females only, and the other (19) reported sex-combined HRs. One study reported the HRs of mortality due to dementia in women only (21).

**Table 1:** Studies included in the qualitative synthesis (*N* = 5).

**First author (year), country**	**Study design**	***N***	**Recruitment**	**Industry**	**Sex**	**Exposure variable**	**Baseline age (mean/SD)**	**Outcome measures**	**Comparison**
Nabe-Nielsen et al. (17), Denmark	Prospective	3,339	Danish national register	General workers	Men and women combined	DW/ SW-NS DW/SW+NS DW/PNS	DW: 45.6 /7.1 SW-NS: 45.1/6.9 SW+NS: 45.4/7.6	Dementia Dx	IRR
Jørgensen et al. (18), Denmark	Prospective	18,015	Danish nurse cohort	Nurse	Women only	DW/ES DW/NS DW/RS	NR	Mortality from Dementia or AD	HR of Mortality
Bokenberger et al. (19), Sweden	Prospective	13,283	STR-1973(national register)	General worker	Men and women combined	Non-SW/SW	Non-SW: 37.8/5.4 SW: 37.7/5.4	Dementia Dx	HR
	Prospective	41,199	SALT cohort (national register)	General worker	Men and women combined	Non-NS/NS	Non-NS: 58.4/10.1 NS: 57.2/10.0	Dementia Dx	HR
Nabe-Nielsen et al. (20), Denmark	Prospective	4,766	Copenhagen male study	employee	Men only	Non-SW/SW Non-LW/LW	Non-SW: 49.1/5.4 SW: 48.4/5.2	Dementia Dx	IRR working hour >45 h
Jørgensen et al. (18), Denmark	Prospective, shift work information in 1993 or 1999	18,892	Danish nurse cohort	Nurse	Women only	DW/ES DW/NS DW/RS	Dementia: 56.1/5.3 Non-dementia: 50.3/4.8	Dementia Dx	HR
	Prospective, shift work information in 1993 and 1999	6,048	Danish nurse cohort	Nurse	Women only	DW/PES DW/PNS DW/PRS DW/Change S between date	Dementia: 58.0/3.9 Non-dementia: 55.7/3.5	Dementia Dx	HR

Dx, diagnosis, HR, hazard ratio; IRR, incidence rate ratio; NR not reported; DW, day work; SW, shift work; NS, night shift; ES, evening shift; RS, rotating shift; LW, long work; S, Shift; P, persistent.

### Results of individual studies

Table 2 presents the studies included in the evaluation of the association between shift work and dementia. One study (19) reported a significant association between shift work and dementia. However, another nurse cohort study revealed a minor association of shift work (including evening, night and rotating shifts) with dementia without statistical significance (18).

**Table 2:** Studies included in the meta-analysis (two studies, four HR for meta-analysis).

**First author (year), country**	**Study design**	***N***	**Recruitment**	**Industry**	**Sex**	**Exposure variable**	**Outcome measures**	**HR**	**95%CI (Low)**	**95%CI (High)**
Bokenberger et al. (19), Sweden	Prospective	41,199	SALT cohort (national register)	General worker	Men and women combined	NS/Non-NS	Dementia Dx	1.12	1.01	1.23
Jørgensen et al. (18), Denmark	Prospective, shift work information in 1993 or 1999	18,892	Danish nurse cohort	Nurse	Women only	ES/DW	Dementia Dx	1.11	0.86	1.42
Jørgensen et al. (18), Denmark	Prospective, shift work information in 1993 or 1999	18,892	Danish nurse cohort	Nurse	Women only	NS/DW	Dementia Dx	1.09	0.81	1.47
Jørgensen et al. (18), Denmark	Prospective, shift work information in 1993 or 1999	18,892	Danish nurse cohort	Nurse	Women only	RS/DW	Dementia Dx	1.23	0.98	1.54

CI confidence interval, Dx diagnosis, HR hazard ratio, NS night shift, DW day work, SW shift work, NW night work, ES evening shift, RS rotating shift.

Both studies performed additional analyses according to shift work duration. Bokenberger et al. (19) classified night shift work according to duration as 1–9 years, 10–19 years, and 20 or more years. Compared with non-night shift workers, shift workers with durations of 1–9 years (HR: 1.1, 95% CI, 0.99–1.28), 10–19 years (HR: 1.13, 95% CI, 0.88–1.28), and 20 or more years (HR: 1.15, 95% CI, 0.96–1.34) had a mildly increased risk of dementia, although without statistical significance. Jørgensen et al. (18) classified shift work (including evening shift, night shift, and rotating shift) according to the duration of 1–5 years and more than 6 years and compared the risk of dementia in these groups with those without shift work experience or shift work duration <1 year. Those who worked night shifts for ≥6 years had a higher risk of dementia than those without a history of shift work or a shift work history of <1 year (HR: 1.46, 95% CI: 1.05–1.63).

### Meta-analysis

**Figure 2:**
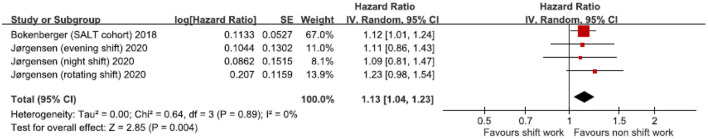
Shift work (SW) and hazard ratio of dementia in the two studies: a random-effect model. CI, confidence interval.

Variations in the association between shift work and dementia were calculated using a random-effect model (Figure 2). The positive trend in pool prevalence was significant (HR = 1.13; 95% CI 1.04 to 1.23; z = 2.85, *p* = 0.04). Low heterogeneity (*I*^2^ = 0%, χ^2^ (2) = 0.64, *P* = 0.89) was also observed.

**Figure 3:**
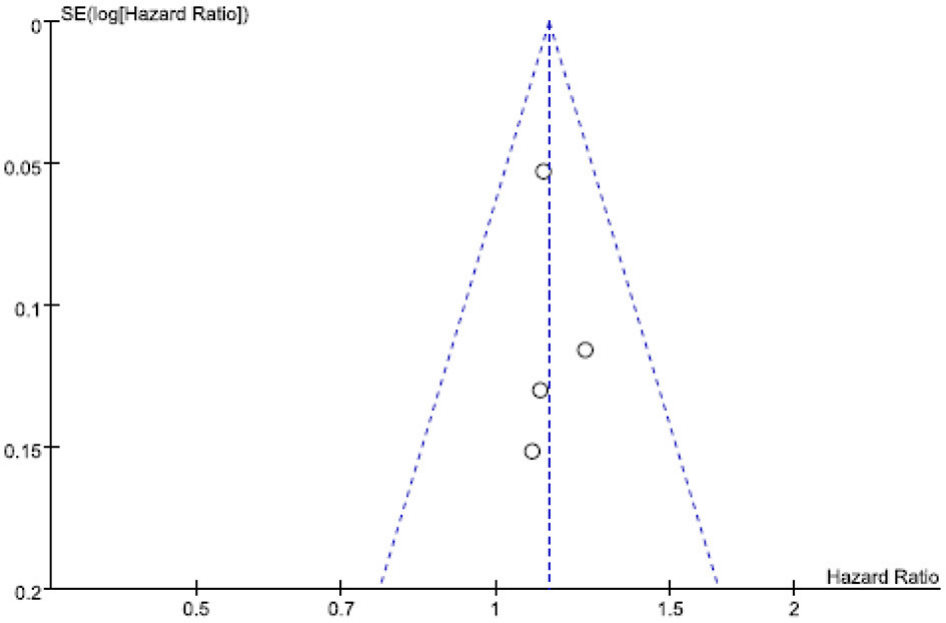
Funnel plot of log-transformed relative risks (RRs) of dementia associated with shift work and standard errors for the two studies.

Figure 3, a funnel plot, shows the log-transformed HRs of dementia associated with shift work, and the SEs showed significant HRs with relatively fewer studies but comparatively smaller SEs (i.e., suitable sizes).

### Subgroup analysis

**Figure 4:**
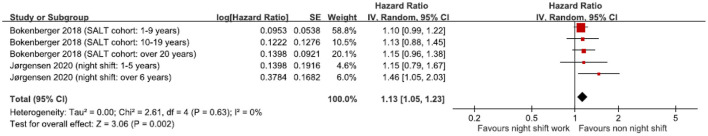
Subgroup analysis of hazard ratio of dementia based on night shift work over 1 year. CI, confidence interval.

We performed a subgroup analysis of those who had worked night shifts for more than 1 year (2 studies generated 5 HRs); the pooled HR was significant (z = 3.06, p = 0.002) at 1.13 (95% CI 1.05 to 1.23) (Table 3, Figure 4). Heterogeneity was low and non-significant (*I*^2^ = 0%, χ^2^ (1) = 2.61, *p* = 0.63).

**Table 3:** Subgroup analysis of hazard ratio based on night shift work for over 1 year.

**Subgroup**	**Hazard ratio**	**95% Confidence interval**
**SALT cohort, Bokenberger** **(****19****)**		
1–9 years	1.10	0.99–1.28
10–19 years	1.13	0.88–1.28
>20 years	1.15	0.96–1.34
**Night SW, Jørgensen et al**. **(****18****)**		
1–5 years	1.15	0.79–1.67
≥6 years	1.46	1.05–2.03
Subtotal	1.13	1.05–1.23

SW, shift work.

## Discussion

To the best of our knowledge, this is the first systematic meta-analysis of the association between dementia and shift work. The estimated pooled HRs of the association between dementia and shift work in the two studies included in the meta-analysis were positive and significant. Both studies had a prospective design and used definite clinical diagnosis of dementia. The samples were from national registrations, and both undertook similar shift work measurements (both collected shift work information, including the duration of shift work, at a single point in time). Some participant characteristics were also similar in these two studies: the baseline age (58.1 ± 10.1 vs. 50.3 ± 4.8 in non-dementia cases and 56.1 ± 5.3 in dementia case), follow-up period (14.1 years vs 12.5 years), and proportion of shift work history (30.1 vs. 37.4%). However, there were some differences between these two studies. One study enrolled people from Denmark, while the other was from Sweden; one enrolled both female and male workers, and the other included only female nurses. Differences in race and sex have been led to different life expectancies (22); however, this did not affect the results much as the above-mentioned studies had similar follow-up times and baseline ages. Due to the low heterogeneity of the included studies, our findings can be considered robust.

Shift work may disturb circadian rhythm, leading to poor sleep quality (23). Several studies revealed that sleep disturbance may increase the risk of developing dementia (24, 25). Currently, some evidence shows several possible underlying mechanisms of circadian dysfunction influencing neurodegeneration. A human study has suggested that sleep disturbance increases the risk of Alzheimer's disease *via* increased amyloid β production (26). Another mouse study also reported that sleep restriction for 6 h/day for 6 weeks by using the modified multiple platform technique, which can eliminate rapid eye movement sleep and reduce slow wave sleep, increased Aβ and pTau accumulation in the brain, correlated with worsened pathological processes of Alzheimer's disease (27). Melatonin, a neurohormone essential for functioning of the clock, was reported be able to reduce the production of amyloid plaques in N2a/APP cells and has the protective effect on Alzheimer's disease (28). However, night-shift workers have decreased melatonin production (29), which might let them prone to develop dementia. Circadian misalignment also results in decreased glucose tolerance, insulin sensitivity and reversion of the cortisol profile. The high cortisol level during initial sleep stage, which is normally with low cortisol level, may cause insulin resistance (30). Recent study reported insulin modulates clearance of Aβ through its effects on lipid metabolism and proteases, and peripheral insulin resistance might precede Aβ accumulation, as the pathological processes of Alzheimer's disease. Furthermore, high level insulin acts as vasoconstrictor, thus insulin resistance related chronic hyperinsulinemia promotes vasoconstriction and results in hypertension which is the most attributed pathophysiology of vascular dementia (31). Besides, it was proposed that circadian clocks in microglia and astrocytes might regulate the blood–brain barrier, inflammation, and synaptic function and might also relate to neurodegeneration if the clock is disrupted (32). And mice with brain-specific deletion of Bmal1, which is known as the master clock gene and regulates redox gene expression and oxidative stress in the brain, developed circadian clock dysfunction, widespread astrocyte activation and synaptic degeneration, also emphasizing the link between the core circadian clock, brain oxidative stress, and neurodegeneration (33, 34).

Several studies have shown that long-term night shift work may have a negative influence on health and is associated with a higher rate of breast cancer, rectal cancer, atrial fibrillation, coronary heart disease, and ischemic stroke (35–39). Our subgroup analysis revealed that undertaking night shift work for more than 1 year was associated higher rate of dementia. Several studies have shown that long-term shift work may have a negative effect on cognition. A prospective cohort study that enrolled 3119 employed and retired workers revealed that shift work was associated with impaired cognition with a stronger association when shift work exceeded 10 years (dose-response effect) (40). Rouch et al. (41) observed that memory performance significantly decreased in men with a 10–20 years shift-work duration. However, other studies have reported conflicting results. Devore et al. found no differences in average cognition for both global and verbal scores between nurses with more than 20 years of shift-work history and nurses with non-shift work history (11). Bokenberger et al. observed that midlife exposure to shift work or night-time work was not associated with significant cognitive changes in verbal, spatial, and memory abilities; processing speed; and general cognitive function in later life (42). According to Marquié et al. (40) cognitive function can recover following cessation of shift work for at least 5 years. Both these studies, however, did not assess whether the participant left shift work or not, which may contribute to the conflict noted in the results of the studies available on this topic.

The current study had several limitations. First, attrition bias may be a major methodological problem in both longitudinal studies, which may affect our results. For example, jobs with night shift work may require more cognitive load than work conducted during normal day shift, and only individuals who can adapt to shift work might tolerate the working style; thus, the self-selection of workers, the health worker effect, may contribute to attrition bias. Second, there was no information on shift work intensity, such as the number of shifts per week/month or hours of work per shift. Third, the studies used national registers as a source of dementia data, which may contribute to bias in terms of the under-detection of dementia, although data from registries may be the most accessible data form for continuous follow-up of participants. Fourth, we could not consider other potential confounders, such as socioeconomic position, nutrition, smoking, and sleep disturbance which might result in the weak association of dementia and shift work in our study. Fifth, given the exposure to light at night, the situation occurred while individuals on the evening shift, night shift or rotating shift may disrupt the circadian rhythms (43), we included people with an ever shift work history to evaluate the association with dementia. However, due to the limited amount of literature in the field, the meta-analysis was based on only two studies. Though the low heterogeneity of the analyzed studies, the pooled HR may be greatly influenced by Bokenberger et al. study (19) and the effects of different types of work schedules is still uncertain in current study, thus future high-quality studies including large sample size with adequate follow up time and exact period of different type of shift work, fixed or rotating shift would be expected to clarify the effect on different type of shift work in dementia. Finally, the included studies enrolled only people in northern Europe; thus, the results cannot be generalized.

## Conclusion

We detected a modest but significant association between shift work and dementia. There was low heterogeneity in the methodologies of the included studies in our meta-analysis. Similarly, subgroup analysis of night work for more than 1 year also revealed a significant association between longer shift work history and risk of dementia. In workplace practice, avoiding long-term night shift work might be effective in reducing the risk of dementia. Further studies are required to confirm this hypothesis.

## Data availability statement

The raw data supporting the conclusions of this article will be made available by the authors, without undue reservation.

## Author contributions

C–CY contributed to the conception and design of the study. K–WL and C–CY contributed to acquisition and drafted the article/revised the article. C–HC and H–YC contributed to analysis. C–HH contributed to interpretation of data. All authors read and approved the final manuscript.
